# Vaccine platform technologies and national security

**DOI:** 10.1186/s42506-021-00070-5

**Published:** 2021-04-20

**Authors:** Mohamed K. M. Khalil

**Affiliations:** 1 Research and Development, Cairo, Egypt; 2 Research and Development, Cairo, Egypt

The risk of global emerging infectious diseases or bioterrorism will remain an important threat to the national security of every country. Coordinated preparedness and response planning across all levels and agencies is an essential strategy in combating cross-border emerging infectious diseases or threats. The COVID-19 pandemic showed more than ever that developing countries can be left behind during the times of need, as political power to access and purchase vaccines and pandemic medications was a crucial factor in the medical countermeasures.

Vaccines are not developed in pharmaceutical companies but in universities and research centers in developed countries [1]. This will keep the majority of developing countries away from the advanced technology of vaccine development like vaccine platforms [2].

Platform vaccine technologies have been developed that could make it possible to shorten the time needed to respond to an emerging infectious disease. Although there is no sharp definition of what constitutes a platform, simply it is a spectrum of technologies that can rapidly produce multiple vaccines from a single pluripotent underlying system or infrastructure [3, 4].

Vaccine platform technology is only an example of how scientific achievements in advanced countries can enhance rapid response to emerging infectious disease threats. Knowledge and technology transfer can bridge the gap to a certain extent between developed and developing countries. However, the localization of advanced vaccine technology needs more than direct investments and strategic partnerships with the leading pharmaceutical companies and research centers. A visionary political commitment to build national expertise in vaccine development is needed. It would allow difficult negotiations and preparedness to be undertaken in a more reasonable timeframe than is possible before or during a pandemic.

Pandemic is not only a threat to an individual country or region. Accordingly, a global movement for technology transfer guided by the WHO or Gavi (Global Alliance for Vaccines and Immunization) is needed to ensure global preparedness and efficient response to an upcoming pandemic. Regional vaccine technology centers may be part of the plan towards a global equal access to vaccines.

## Data Availability

Not applicable.

## Abbreviations

COVID-19: Coronavirus disease 2019
WHO: World Health Organization
Gavi: Vaccine Alliance—Global Alliance for Vaccines and Immunization
